# Epidemiological and clinical characteristics of injuries among adolescents attended at an emergency service in the city of Ribeirão Preto, São Paulo

**DOI:** 10.1590/S1516-31802012000100005

**Published:** 2012-02-13

**Authors:** Luiz Antonio Del Ciampo, Ivan Savioli Ferraz, Maria Tazima, Letícia Graziela Bachette, Karla Ishikawa, Rodrigo Paixão

**Affiliations:** I MD, PhD. Professor, Department of Childcare and Pediatrics, Faculdade de Medicina de Ribeirão Preto (FMRP), Universidade de São Paulo (USP), Ribeirão Preto, São Paulo, Brazil.; II MD, PhD. Professor, Department of Childcare and Pediatrics, Faculdade de Medicina de Ribeirão Preto (FMRP), Universidade de São Paulo (USP), Ribeirão Preto, São Paulo, Brazil.; III MD, PhD. General Surgeon and Professor, Division of Pediatric Surgery, Department of Surgery and Anatomy, Faculdade de Medicina de Ribeirão Preto (FMRP), Universidade de São Paulo (USP), Ribeirão Preto, São Paulo, Brazil.; IV Student, Department of Childcare and Pediatrics, Faculdade de Medicina de Ribeirão Preto (FMRP), Universidade de São Paulo (USP), Ribeirão Preto, São Paulo, Brazil.; V Student, Department of Childcare and Pediatrics, Faculdade de Medicina de Ribeirão Preto (FMRP), Universidade de São Paulo (USP), Ribeirão Preto, São Paulo, Brazil.; VI Student, Department of Childcare and Pediatrics, Faculdade de Medicina de Ribeirão Preto (FMRP), Universidade de São Paulo (USP), Ribeirão Preto, São Paulo, Brazil.

**Keywords:** Accidents, Adolescent, Morbidity, Epidemiology, Adolescent health services, Acidentes, Adolescente, Morbidade, Epidemiologia, Serviços de saúde para adolescentes

## Abstract

**CONTEXT AND OBJECTIVE:**

Injuries are an important cause of morbidity during adolescence, but can be avoided through learning about some of their characteristics. This study aimed to identify the most frequent injuries among adolescents attended at an emergency service.

**DESIGN AND SETTING:**

Retrospective descriptive study on adolescents attended at the emergency service of the Teaching Health Center, Faculdade de Medicina de Ribeirão Preto (FMRP), between January 1, 2009, and September 30, 2009.

**METHODS:**

Age, sex, type of injury, site, day and time of occurrence, part of body involved, care received, whether the adolescent was accompanied at the time of injury and whether any type of counseling regarding injury prevention had been given were analyzed.

**RESULTS:**

Among 180 adolescents attended, 106 (58.8%) were boys and 74 (41.1%) were girls. Their ages were: 10 to 12 (66/36.6%), 12 to 14 (60/33.3%) and 14 to 16 years (54/30%). The injuries had occurred in public places (47.7%) and at home (21.1%). The main types were bruises (45.1%) and falls (39.2%), involving upper limbs (46.1%), lower limbs (31%) and head/neck (13.1%). The injuries occurred in the afternoon (44.4%) and morning (30%), on Mondays (17.7%) and Thursdays (16.6%). Radiological examinations were performed on 53.8%. At the time of injury, 76.1% of the adolescents were accompanied. Some type of counseling about injury prevention had been received by 39.4%.

**CONCLUSIONS:**

Although the injuries were of low severity, preventive attitudes need to be incorporated in order to reduce the risks and provide greater safety for adolescents.

## RESUMO

**CONTEXTO E OBJETIVO:**

Lesões são importante causa de morbidade na adolescência, mas podem ser evitadas mediante o conhecimento de algumas de suas características. Este estudo visa identificar as lesões mais frequentes entre adolescentes atendidos em um serviço de pronto-atendimento.

**TIPO DE ESTUDO E LOCAL:**

Estudo retrospectivo e descritivo sobre adolescentes atendidos no Pronto-Atendimento do Centro de Saúde Escola da Faculdade de Medicina de Ribeirão Preto (FMRP), entre 01/01/2009 e 30/9/2009.

**MÉTODOS:**

Analisou-se idade, sexo, tipo de lesão, local, dia e período do ocorrido, parte do corpo acometida, cuidados recebidos, se o adolescente estava acompanhado no momento da lesão e se havia recebido alguma orientação quanto à prevenção de acidentes.

**RESULTADOS:**

Dos 180 adolescentes 106 (58,8%) eram rapazes e 74 (41,1%) moças, com idades entre 10 a 12 anos (66/36,6%), 12 a 14 anos (60/33,3%) e 14 a 16 anos (54/30%). 47,7% das lesões aconteceram em locais públicos e 21,1%, em casa. Os principais foram contusões (45,1%) e quedas (39,2%), acometendo membros superiores (46,1%), membros inferiores (31%) e cabeça/pescoço (13,1%). As lesões ocorreram nos períodos da tarde (44,4%) e manhã (30%) e às segundas-feiras (17,7%) e quintas-feiras (16,6%). Foram realizados exames radiológicos em 53,8% dos pacientes. No momento da lesão 76,1% dos adolescentes estavam acompanhados. 39,4% haviam recebido algum tipo de orientação sobre prevenção de lesões.

**CONCLUSÕES:**

Embora as lesões tenham sido de baixa gravidade, é necessário incorporar atitudes preventivas de modo a diminuir os riscos e proporcionar maior segurança aos adolescentes.

## INTRODUCTION

In the 21^st^ century, injuries continue to be an important cause of morbidity, permanent disability and death over various age ranges. Worldwide, more than 2.5 million people aged 10 to 24 years die every year as victims of injuries.^1^^,^^2^ It has been estimated that by the year 2030, mortality due to injuries will increase by about 40%, especially due to traffic accidents and the improved socioeconomic conditions of the population.^3^ Among adolescents, injuries are the main cause of mortality in the age range of 15 to 19 years.^4^ In the United States, injuries are responsible for more adolescent deaths than all the other causes taken together. In Brazil, in 2004, 69.5% of adolescent deaths were due to injuries and in 2005, external causes were responsible for 9.4% of the hospitalizations of patients aged 10 to 19 years.^5^

Every year, almost 25% of all adolescents suffer some type of serious injury that requires specialized medical care and limits the activities of the victims.^6^ A review found that for each death due to trauma, 15 other adolescents were severely affected by injuries, more than 640 were hospitalized and about another 40 suffered lesions requiring long-term follow-up and rehabilitation treatment.^2^

In addition to the immediate physical problems, injuries can lead to great economic losses, represented by absences from work and school. Additional emotional and social costs are involved, affecting victims and their parents, when the potential years of life lost are taken into consideration. Such losses deprive society of the intellectual and economic potential of the injured individuals.^7^^,^^8^ Adolescents who suffer physical or emotional sequelae of injuries will also definitely suffer impairment of their body image, which will greatly influence their quality of life.

Epidemiological studies on health problems due to external causes are important because they make it possible to recognize patterns of events and identify at-risk populations. This can help in allocating resources and structuring services, in addition to contributing towards planning of healthcare activities and determining the feasibility of appropriate strategies.^8^^,^^9^^,^^10^

## OBJECTIVE

The aim of the present study was to identify the most frequent injuries suffered by adolescents attended at an emergency service and the characteristics of such injuries.

## METHODS

This was a retrospective descriptive study based on medical record data relating to all adolescents who had suffered injuries and were attended at the emergency service of Centro de Saúde-Escola (CSE) “Joel Domingos Machado”, Faculdade de Medicina de Ribeirão Preto (FMRP), Universidade de São Paulo (USP), between January 1, 2009, and September 30, 2009. This emergency care service is open daily, 24 hours/day, and provides a reference health service for approximately 150,000 inhabitants of the city of Ribeirão Preto. Patients come to the emergency service spontaneously or are brought in by the mobile emergency care service.

From the medical records, the victim’s age and sex were ascertained, along with event information such as type of accident, place of occurrence, date and time of the incident, affected body part, care received at the health service, whether the adolescent was accompanied at the time of the accident and by whom, and whether any guidance on how to prevent accidents (at home, school, service, health care, community) had previously been given.

Accidents were defined as any cases for which the diagnosis is contained in Chapter XIX of the International Classification of Diseases, 10^th^ revision (Injuries, Poisoning and Certain Consequences of External Causes: S00 to T98). This research project was assessed and approved by the Research Ethics Committee of CSE-FMRP-USP.

## RESULTS

Between January 1, 2009, and September 30, 2009, the emergency care service attended 6,614 adolescents and, of these, 180 cases (2.7%) were seen because of injuries. These cases comprised 106 males (58.8%) and 74 females (41.1%), distributed in the age ranges of 10 to 12 years (66/36.6%), 12 to 14 years (60/33.3%) and 14 to 16 years (54/30%). 47.7% of the injuries occurred in public places, mainly in the streets, 31.1% at school and 21.1% at home. At the time of the injury, 76.1% of the adolescents were accompanied by friends, as shown in Table 1.

**Table 1.** Distribution of some characteristics of injured adolescents and their accidents

	n	%
Sex		
Male	106	58.5
Female	74	41.1
Age (years)		
10 to 12	66	36.6
12 to 14	60	33.3
14 to 16	54	30.0
Place of accident		
Street	72	40
School	56	31.1
Home	43	23.8
Accompanied		
Yes	137	76.1
No	43	23.8
Previous counseling		
Yes	71	39.4
No	109	60.5

Tables 2 and 3 list other characteristics of the adolescents and the events. The main types of injuries were physical collisions against obstacles, i.e. walls, doors, furniture and objects (45.1%), and falls (39.2%). The body parts most frequently involved were the upper limbs (46.1%), lower limbs (31%) and head/neck region (13.1%). Guidelines about injury prevention had already been given to 39.4% of the patients, who reported that they had obtained them at primary healthcare units (39.1%), at home with their families (25%), through the media (18.4%) and at school (17.4%).

No deaths or cases requiring hospitalization were recorded. All the patients were discharged, although some (7.7%) were later referred to specialist professionals such as orthopedists, neurologists or physiotherapists.

The injuries occurred in the afternoon (44.4%) and morning (30%), on Mondays (17.7%) and Thursdays (16.6%). Radiological examinations were performed on 53.8%.

**Table 2.** Distribution of the types of injuries

Type of injury	n	%
Physical collision	84	45.1
Fall	73	39.2
Sting by venomous animals	12	6.4
Cut	10	5.3
Burn	3	1.6
Animal bite	2	1.0
Drug intoxication	2	1.0
Total	186	100

**Table 3.** Distribution of injuries according to the body region involved

Body region involved	n	%
Upper limbs	95	46.1
Lower limbs	64	31.0
Head and neck	27	13.1
Chest	14	6.79
Abdomen	6	2.91
Total	206	100

## DISCUSSION

The predominance of injury occurrences among males has been observed in many studies and can be explained by the life dynamics and activities performed by boys, which expose them to greater risk of suffering injuries.^11^^,^^12^Among the adolescents attended at emergency services in the city of Recife, Pernambuco, between January 2004 and December 2005^13^ and in the city of Ipatinga, Minas Gerais,^14^ consequent to injuries, males were more frequently involved.

In the present study, physical collisions against obstacles, bruises and falls were the injuries most commonly recorded. This finding is in agreement with other studies, such as the one conducted on children and adolescents attended at an emergency service in the city of Londrina, Paraná, which revealed that falls were the most prevalent accidents (33.9%), while the injuries more frequently observed were bruises due to superficial trauma (22%) and fractures (19.5%).^15^ Other authors have also observed that falls (33.2%) were the most prevalent accidents.^14^ In the city of Belém, Pará, a study on 2,828 adolescents aged 17 and 18 years revealed that falls (35%), cuts (30%), burns (22%) and bites (18%) were the injuries most commonly found, with a higher frequency among males.^5^ Falls, collisions, burns and intoxications were also the injuries most frequently observed among children and adolescents under the age of 15 years in the United States, thus demonstrating that injuries occur in all environments, regardless of the victims’ socioeconomic conditions.^16^

Fractures are also an important consequence of accidental injuries.^4^ The risk of fractures before the age of 16 years is approximately 40% among boys and 5% among girls, most frequently involving the distal region of the forearm and the hand.^17^ A study on 10,203 Swedish adolescents found that fractures of the distal forearm caused by falls were the most common type, and that the rate of occurrence was 1.5 times higher among boys.^18^

As expected, injuries occurred more outside the home, especially in public places such as streets (47.7%) and at school (31.1%), since adolescents tend to spend most of their time in activities outside of their homes, preferentially in the company of friends. Among American adolescents, almost 25% of the injuries occurred at school, more than half of them during sports activities, and were twice as common among boys. The streets are considered to be a high risk place for injuries, since traffic is responsible for about one third of all deaths.^2^

According to the World Health Organization, nearly 45% of the injuries affecting the world’s population occur at home. In England, practically half of all fatal injuries occur at home, and in the United States, one third of all accidental injuries and nearly 25% of all deaths caused by accidents occur within domestic environments, with yearly losses of 117 billion United States dollars.^19^

The involvement of the upper limbs (46.1%) and lower limbs (31.0%) observed in the present study was directly related to defense and protection movements and reflexes and to practicing of radical physical activities, since adolescents have already reached a more evolved stage of motor maturity. A study on children and adolescents who were victims of traffic accidents and were admitted to a tertiary care public hospital in the city of Fortaleza, Ceará, revealed predominance of male victims (73.3%) and involvement of the head and limbs.^20^ Similar results were obtained in the United States, in a study on hospital attendance of children and adolescents consequent to injuries, which found that 62.4% of the cases were boys, mainly presenting injuries to the head (29%) and upper limbs (20.6%).^21^

The present study revealed that 74% of the injuries occurred during the daytime, and practically one third of them occurred during weekends. These findings can be interpreted as being due to the varied activities in which adolescents engage, especially with regard to sports practices, which represent daily situations predisposing towards injuries. This was also reported by other authors who observed that, through practicing sports activities for approximately 14 hours per month, on average, adolescents end up being more exposed to acute traumatic injuries.^22^

Surveillance and studies using epidemiological data associated with accidents are important elements in controlling injury rates.^23^ Pediatric practitioners and physicians in general have a responsibility to invest in educational process for patients and their relatives, in addition to demystifying the notion that accidents are simply random occurrences, given that they can and must be avoided. They also have a duty to propose environmental improvements, risk factor controls and changes to the legislation.^4^^,^^19^^,^^23^^,^^24^

## CONCLUSIONS

The results from this study identified that bruises and falls were the injuries that occurred most often. They occurred predominantly among boys aged 10 to 12 years, in public places. Since behavior during adolescence may represent a phase of exposure to risks, the topic of injuries should be included in prevention programs directed towards adolescents. The intention of the present study was to contribute to the expansion of information about the characteristics of injuries and their victims. Nevertheless, the limitations of this study on data collected over a period of only nine months need to be recognized.
